# Supplementary material for: The Association between Gut Microbiota and Uremia of Chronic Kidney Disease
Source: Microorganisms. 2020 Jun 16;8(6):907. doi: 10.3390/microorganisms8060907 (PMC7355700; doi:10.3390/microorganisms8060907)
Supplement: Supplementary file 1 [file microorganisms-08-00907-s001.pdf]

Supplementary materials

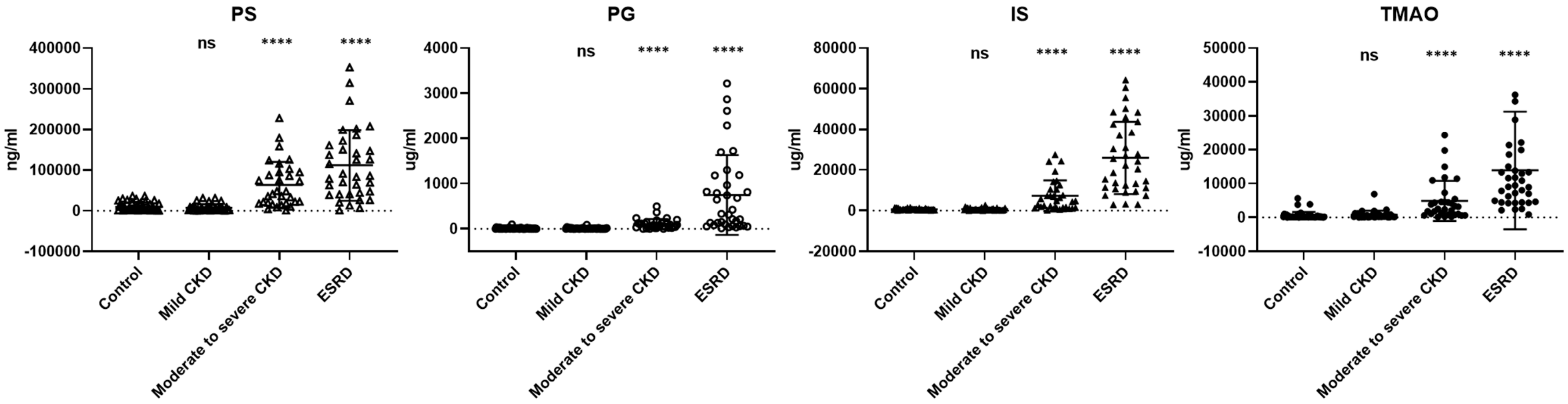

Figure S1. Serum levels of four uremic metabolites according to CKD groups. The p-values of multiple comparison in each CKD group compared to control are plotted on the graph (ns, not significant; ****, p<0.001). Abbreviations: PS, P-cresyl sulfate; PG, P-cresyl glucuronide; IS, indoxyl sulfate; TMAO, Trimethylamine N-oxide; CKD, chronic kidney disease; ESRD, end stage renal disease.

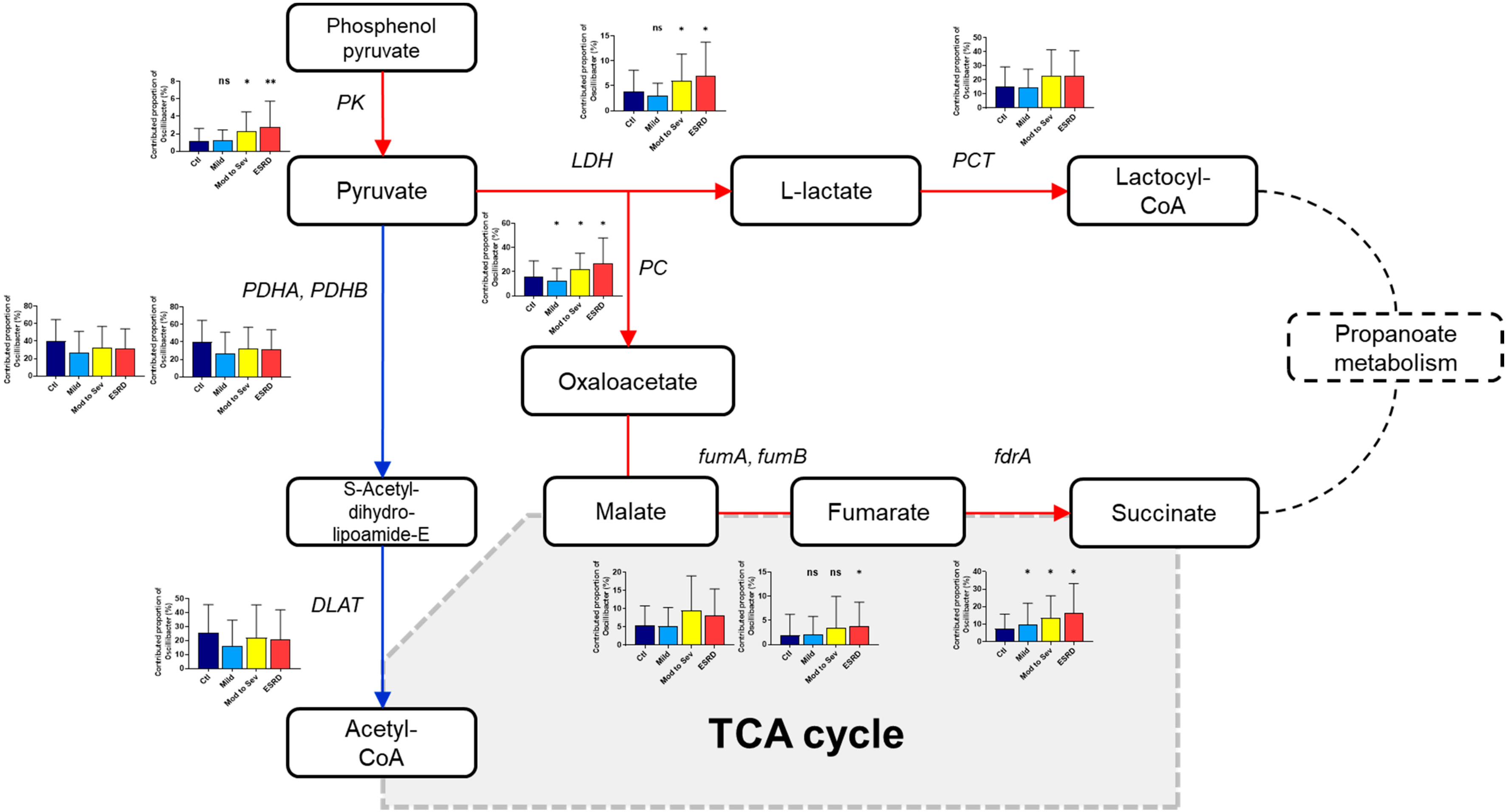

Figure S2. Hypothetical pathway diagram using the KEGG module associated with changes in *Oscillibacter* contribution according to the renal function. The red and blue arrows represent schematic pathways that increase or decrease the contribution of *Oscillibacter* as renal function deteriorates, respectively. Small bar charts represent comparison of contribution of *Oscillibacter* in each orthlogs by groups. Asterisks in bar charts indicate significance (q-value) compared to control group. ns, not significant; *, q<0.05; **, q<0.005. Abbreviation: KEGG, Kyoto Encyclopedia of Genes and Genomes.

Table S1. Differentially represented KEGG categories predicted by PICRUSt (level 3) in 4 groups.

Level_1	Level_2	Level_3	p-values*	p-values* (corrected)
Metabolism	Metabolism of Cofactors and Vitamins	Riboflavin metabolism	5.93E-05	0.019438
Metabolism	Carbohydrate Metabolism	Pyruvate metabolism	0.000219	0.02393
Metabolism	Energy Metabolism	Methane metabolism	0.000153	0.025037
Metabolism	Others	Others_Unclassified	0.000372	0.030475
Metabolism	Biosynthesis of Other Secondary Metabolites	Flavonoid biosynthesis	0.00109	0.051083
Metabolism	Metabolism of Cofactors and Vitamins	Nicotinate and nicotinamide metabolism	0.001352	0.055414
Metabolism	Nucleotide Metabolism	Purine metabolism	0.001539	0.05607
Metabolism	Xenobiotics Biodegradation and Metabolism	Chloroalkane and chloroalkene degradation	0.001039	0.056804
Genetic Information Processing	Translation	Ribosome biogenesis in eukaryotes	0.000947	0.062124
Cellular Processes	Cell Growth and Death	Apoptosis	0.001973	0.064699
Metabolism	Biosynthesis of Other Secondary Metabolites	Isoquinoline alkaloid biosynthesis	0.002609	0.077794
Metabolism	Lipid Metabolism	Glycerophospholipid metabolism	0.003932	0.085988
Metabolism	Carbohydrate Metabolism	Glycolysis / Gluconeogenesis	0.003918	0.091784
Metabolism	Metabolism of Terpenoids and Polyketides	Tetracycline biosynthesis	0.005625	0.092257
Human Diseases	Infectious Diseases	Amoebiasis	0.00342	0.093481
Metabolism	Energy Metabolism	Photosynthesis proteins	0.004608	0.094461
Metabolism	Energy Metabolism	Photosynthesis	0.005498	0.094917
Metabolism	Metabolism of Cofactors and Vitamins	Folate biosynthesis	0.006179	0.096513

Metabolism	Carbohydrate metabolism	Carbohydrate metabolism_Unclassified	0.003827	0.096552
Metabolism	Lipid Metabolism	Arachidonic acid metabolism	0.005369	0.097833
Metabolism	Glycan Biosynthesis and Metabolism	Lipopolysaccharide biosynthesis	0.005158	0.099522
Organismal Systems	Digestive System	Carbohydrate digestion and absorption	0.007076	0.105495
Metabolism	Glycan Biosynthesis and Metabolism	Various types of N-glycan biosynthesis	0.00766	0.109233
Genetic Information Processing	Translation	Ribosome Biogenesis	0.009099	0.114785
Metabolism	Metabolism of Other Amino Acids	Phosphonate and phosphinate metabolism	0.008816	0.11567
Metabolism	Amino Acid Metabolism	Valine, leucine and isoleucine biosynthesis	0.008553	0.116888
Metabolism	Lipid Metabolism	Glycerolipid metabolism	0.013993	0.120781
Cellular Processes and Signaling	Other transporters	Other transporters_Unclassified	0.015123	0.120983
Metabolism	Amino acid metabolism	Amino acid metabolism_Unclassified	0.011816	0.121111
Metabolism	Xenobiotics Biodegradation and Metabolism	Xylene degradation	0.013827	0.122576
Metabolism	Metabolism of Terpenoids and Polyketides	Carotenoid biosynthesis	0.014993	0.122944
Genetic Information Processing	Replication and Repair	Chromosome	0.011691	0.123698
Metabolism	Biosynthesis of Other Secondary Metabolites	Tropane, piperidine and pyridine alkaloid biosynthesis	0.014779	0.124298
Metabolism	Xenobiotics Biodegradation and Metabolism	Styrene degradation	0.012912	0.124566
Metabolism	Amino Acid Metabolism	Histidine metabolism	0.01265	0.125734
Metabolism	Carbohydrate Metabolism	Pentose and glucuronate interconversions	0.013812	0.125841
Metabolism	Glycan Biosynthesis and Metabolism	Glycosyltransferases	0.011606	0.126895

Cellular Processes	Cell Growth and Death	Meiosis - yeast	0.013611	0.127555
Metabolism	Amino Acid Metabolism	Arginine and proline metabolism	0.01129	0.127692
Genetic Information Processing	Folding, Sorting and Degradation	RNA degradation	0.019537	0.130781
Metabolism	Xenobiotics Biodegradation and Metabolism	Naphthalene degradation	0.01883	0.131412
Metabolism	Xenobiotics Biodegradation and Metabolism	Dioxin degradation	0.018059	0.13163
Metabolism	Lipid Metabolism	Secondary bile acid biosynthesis	0.019291	0.131824
Metabolism	Xenobiotics Biodegradation and Metabolism	Bisphenol degradation	0.017715	0.132056
Metabolism	Lipid Metabolism	Steroid biosynthesis	0.011279	0.132124
Metabolism	Lipid Metabolism	Primary bile acid biosynthesis	0.018647	0.132958
Metabolism	Energy Metabolism	Carbon fixation in photosynthetic organisms	0.01095	0.133021
Metabolism	Metabolism of Other Amino Acids	Selenocompound metabolism	0.017612	0.134343
Metabolism	Biosynthesis of Other Secondary Metabolites	beta-Lactam resistance	0.017336	0.135387
Metabolism	Metabolism of Terpenoids and Polyketides	Terpenoid backbone biosynthesis	0.023775	0.144409
Metabolism	Biosynthesis of Other Secondary Metabolites	Caffeine metabolism	0.022059	0.144709
Genetic Information Processing	Transcription related proteins	Transcription related proteins_Unclassified	0.022615	0.145442
Metabolism	Enzyme Families	Protein kinases	0.02444	0.145748
Environmental Information Processing	Signaling Molecules and Interaction	Cellular antigens	0.023595	0.146019
Metabolism	Lipid Metabolism	Linoleic acid metabolism	0.026903	0.147068
Human Diseases	Infectious Diseases	Vibrio cholerae pathogenic cycle	0.02518	0.147481

Metabolism	Metabolism of Cofactors and Vitamins	Ubiquinone and other terpenoid-quinone biosynthesis	0.023419	0.147719
Environmental Information Processing	Signal Transduction	Phosphatidylinositol signaling system	0.02674	0.148657
Human Diseases	Neurodegenerative Diseases	Alzheimer's disease	0.027697	0.148929
Metabolism	Metabolism of Terpenoids and Polyketides	Prenyltransferases	0.026729	0.151157
Metabolism	Metabolism of Terpenoids and Polyketides	Zeatin biosynthesis	0.029528	0.151329
Organismal Systems	Digestive System	Protein digestion and absorption	0.030072	0.151748
Metabolism	Glycan Biosynthesis and Metabolism	N-Glycan biosynthesis	0.028708	0.151876
Genetic Information Processing	Folding, Sorting and Degradation	Chaperones and folding catalysts	0.029295	0.152521
Metabolism	Lipid Metabolism	Fatty acid elongation in mitochondria	0.026607	0.153109
Genetic Information Processing	Transcription	Basal transcription factors	0.033423	0.158882
Metabolism	Biosynthesis of Other Secondary Metabolites	Phenylpropanoid biosynthesis	0.034105	0.159805
Organismal Systems	Environmental Adaptation	Circadian rhythm - plant	0.032382	0.160927
Metabolism	Metabolism of Other Amino Acids	D-Arginine and D-ornithine metabolism	0.033405	0.161128
Metabolism	Amino Acid Metabolism	Amino acid related enzymes	0.033037	0.161734
Genetic Information Processing	Translation	Ribosome	0.037007	0.164032
Metabolism	Glycan Biosynthesis and Metabolism	Lipopolysaccharide biosynthesis proteins	0.036558	0.164259
Cellular Processes and Signaling	Germination	Germination_Unclassified	0.036539	0.166456
Organismal Systems	Excretory System	Proximal tubule bicarbonate reclamation	0.036517	0.168696

Metabolism	Carbohydrate Metabolism	Pentose phosphate pathway	0.041547	0.170343
Cellular Processes and Signaling	Cell division	Cell division_Unclassified	0.040196	0.171226
Metabolism	Carbohydrate Metabolism	Ascorbate and aldarate metabolism	0.041241	0.17123
Organismal Systems	Immune System	RIG-I-like receptor signaling pathway	0.039921	0.172291
Metabolism	Xenobiotics Biodegradation and Metabolism	Nitrotoluene degradation	0.042549	0.172299
Metabolism	Metabolism of Cofactors and Vitamins	Vitamin B6 metabolism	0.041047	0.172608
Genetic Information Processing	Translation	Aminoacyl-tRNA biosynthesis	0.039696	0.173605
Metabolism	Nucleotide Metabolism	Pyrimidine metabolism	0.046757	0.174275
Human Diseases	Infectious Diseases	Tuberculosis	0.044856	0.175151
Human Diseases	Immune System Diseases	Primary immunodeficiency	0.046517	0.175376
Environmental Information Processing	Signal Transduction	MAPK signaling pathway - yeast	0.046053	0.175646
Environmental Information Processing	Signaling Molecules and Interaction	Bacterial toxins	0.045832	0.176856
Environmental Information Processing	Signaling Molecules and Interaction	Ion channels	0.044819	0.177115
Genetic Information Processing	Translation	RNA transport	0.044316	0.177263

* *p*-values were calculated by Kruskal-Wallis test and corrected by Benjamini-Hochberg method.

Table S2. Contribution of *Oscillibacter* to functional orthologs associated with the pyruvate metabolism pathway.

Functional orthologs	Enzymes	gene	Mean contribution (%) in total sample	Mean contribution (%)				Kruskal-Wallis P-value
				Control	Mild CKD	Moderate to Severe CKD	ESRD	

K00162	pyruvate dehydrogenase E1 component beta subunit	PDHB, pdhB	33.1	40.2	27.0	32.2	31.4	0.107
K00161	pyruvate dehydrogenase E1 component alpha subunit	PDHA, pdhA	33.0	40.1	26.9	32.2	31.4	0.108
K00171	pyruvate ferredoxin oxidoreductase delta subunit	porD	28.2	26.2	28.5	30.2	28.6	0.989
K00170	pyruvate ferredoxin oxidoreductase beta subunit	porB	21.6	19.6	21.4	23.2	22.9	0.914
K00627	pyruvate dehydrogenase E2 component (dihydrolipoamide acetyltransferase)	DLAT, aceF, pdhC	21.3	25.3	16.1	22.0	21.0	0.210
K00169	pyruvate ferredoxin oxidoreductase alpha subunit	porA	19.9	17.5	19.2	21.5	22.0	0.865
K00172	pyruvate ferredoxin oxidoreductase gamma subunit	porG	19.6	17.5	18.5	21.0	21.9	0.873
K01958	pyruvate kinase	PK, pyk	18.7	15.5	12.1	21.7	26.6	<0.001
K01026	propionate CoA-transferase	pct	18.4	14.9	14.4	23.1	22.6	0.031
K15024	putative phosphotransacetylase	K15024	14.5	12.6	12.2	18.2	15.9	0.236
K01069	hydroxyacylglutathione hydrolase	gloB, gloC, HAGH	14.0	12.1	10.1	16.6	18.0	0.081
K01067	acetyl-CoA hydrolase	ACH1	12.8	7.6	7.6	14.1	21.8	0.003
K01679	fumarate hydratase, class II	fumC, FH	12.0	10.4	8.3	13.8	15.8	0.077
K00128	aldehyde dehydrogenase (NAD+)	ALDH	11.7	10.7	9.3	12.8	14.3	0.247
K00244	fumarate reductase flavoprotein subunit	frdA	11.4	7.5	9.6	13.5	16.1	0.005
K00626	acetyl-CoA C-acetyltransferase	atoB	8.0	6.4	5.7	10.1	10.2	0.029
K01962	acetyl-CoA carboxylase carboxyl transferase subunit alpha	accA	7.9	5.7	6.4	10.9	9.3	0.075
K02160	acetyl-CoA carboxylase biotin carboxyl carrier protein	accB, bccP	7.8	6.1	5.9	10.3	9.5	0.090

K01677	fumarate hydratase subunit alpha	fumA	6.8	5.3	5.1	9.5	8.0	0.033
K00027	malate dehydrogenase (oxaloacetate-decarboxylating)	ME2, sfcA, maeA	6.8	5.8	5.0	8.8	8.1	0.067
K01963	acetyl-CoA carboxylase carboxyl transferase subunit beta	accD	6.6	5.0	5.3	8.9	7.7	0.099
K00016	L-lactate dehydrogenase	LDH, ldh	4.8	3.7	3.0	6.0	6.9	0.003
K04072	acetaldehyde dehydrogenase / alcohol dehydrogenase	adhE	4.7	4.0	3.6	6.2	5.6	0.315
K03737	acetate kinase	ackA	4.4	3.1	2.8	5.9	6.3	<0.001
K01571	oxaloacetate decarboxylase (Na+ extruding) subunit alpha	oadA	4.2	3.8	2.9	5.6	4.6	0.141
K00382	dihydrolipoamide dehydrogenase	DLD, lpd, pdhD	3.4	2.4	2.1	4.3	5.1	0.001
K01961	acetyl-CoA carboxylase, biotin carboxylase subunit	accC	2.9	2.3	2.1	3.5	4.0	0.011
K01678	fumarate hydratase subunit beta	fumB	2.8	1.9	2.1	3.5	3.7	0.032
K01006	pyruvate, orthophosphate dikinase	ppdK	2.7	1.5	1.5	4.4	3.7	<0.001
K01759	lactoylglutathione lyase	GLO1, gloA	2.5	2.0	1.7	3.1	3.6	0.011
K00625	phosphate acetyltransferase	pta	2.5	1.5	1.4	3.4	3.9	<0.001
K01649	2-isopropylmalate synthase	leuA, IMS	2.0	1.6	1.4	2.4	2.8	0.012
K00925	pyruvate carboxylase	PC, pyc	1.9	1.3	1.2	2.3	2.9	<0.001
K00873	pyruvate-ferredoxin/flavodoxin oxidoreductase	por, nifJ	1.8	1.2	1.2	2.3	2.8	<0.001
K00656	formate C-acetyltransferase	pflD	1.4	1.1	1.0	1.8	1.9	0.009
K00174	2-oxoglutarate/2-oxoacid ferredoxin oxidoreductase subunit alpha	korA, oorA, oforA	1.3	0.8	0.7	1.8	2.1	0.003
K00175	2-oxoglutarate/2-oxoacid ferredoxin oxidoreductase subunit beta	korB, oorB, oforB	1.3	0.8	0.7	1.7	2.0	0.003
